# Molecular Drivers of Vascular Adaptation in Young Athletes: An Integrative Analysis of Endothelial, Metabolic and Lipoprotein Biomarkers

**DOI:** 10.3390/biom15121726

**Published:** 2025-12-11

**Authors:** Jonas Haferanke, Lisa Baumgartner, Maximilian Dettenhofer, Stefanie Huber, Frauke Mühlbauer, Tobias Engl, Paulina Wasserfurth, Karsten Köhler, Renate Oberhoffer, Thorsten Schulz, Sebastian Freilinger

**Affiliations:** 1 Institute of Preventive Pediatrics, Department Health and Sport Sciences, TUM School of Medicine and Health, Technical University of Munich (TUM), 80809 Munich, Germany; 2 Associate Professorship of Exercise, Nutrition and Health, Department Health and Sport Sciences, TUM School of Medicine and Health, Technical University of Munich (TUM), 80809 Munich, Germany

**Keywords:** adolescents, arterial stiffness, cardiovascular adaptation, endothelin-1, exercise, free triiodothyronine, high-density lipoprotein, leptin, low-density lipoprotein, nitric oxide

## Abstract

Adolescence is a critical window for cardiovascular (CV) development, yet the molecular drivers of vascular adaptation to regular exercise in youth remain poorly understood. This cross-sectional study assessed vascular structure and function alongside endothelial, metabolic, and lipoprotein biomarkers in 203 healthy young athletes (aged 10–16). Vascular phenotyping included carotid intima-media thickness (IMT), pulse wave velocity, and carotid deformation indices (strain, strain rate). Circulating nitric oxide (NO), endothelin-1, free triiodothyronine (fT3), leptin, low-density lipoprotein, and high-density lipoprotein were analyzed. Associations were examined using hierarchically adjusted multivariable linear regression, mediation and moderation were tested and sex-stratified/matched analyses were conducted. While training volume was not associated with endothelial markers, leptin was correlated positively with NO and negatively with diastolic strain rate, suggesting dual vascular actions. fT3 was inversely associated with IMT, indicating a potential protective role in vascular remodeling. Lipoprotein profiles showed no independent associations with vascular parameters. Hemodynamic load, particularly systolic blood pressure, emerged as the dominant determinant of arterial stiffness. Sex-specific differences across biomarkers and vascular indices support a multifactorial model: in active youth, vascular phenotype reflects hemodynamics, body composition, and endocrine–metabolic signals more than training; longitudinal mechanistic studies should clarify causal pathways and guide individualized cardiovascular risk profiling.

## 1. Introduction

Childhood and adolescence constitute critical periods for establishing lifelong cardiovascular (CV) health, as major risk factors often arise early in life [1,2]. Across these life stages, the CV system undergoes substantial structural and functional maturation [3], and adequate development is associated with reduced lifetime risk of atherosclerosis, hypertension, and coronary artery disease [4,5,6].

Regular PA and exercise in youth confer robust benefits such as enhanced cardiorespiratory fitness, body composition, and overall psychosocial well-being, collectively lowering future morbidity and mortality [7,8,9]. Although PA and exercise are conceptually distinct, from a CV perspective they elicit largely overlapping adaptations: Mechanistically, it enhances endothelial function [10,11], optimizes blood pressure [12], regulates lipid metabolism [13,14], and mitigates inflammation [15] and oxidative stress [16]. Accordingly, physically active youth are an informative cohort for lifestyle-driven modulation of CV maturation.

In this sense, public health guidelines by the World Health Organization recommend at least 60 min of moderate-to-vigorous physical activity daily for children and adolescents [17]. Competitive young athletes commonly exceed this recommendation with high training volumes and frequent competition [18,19]. Although such exposures elicit distinct CV adaptations, their magnitude and clinical significance remain unclear [20,21]. Studies report heterogenous cardiac remodeling [22] and vascular changes in youth athletes, including alterations in arterial elasticity and intima-media thickness (IMT) [23], with inconsistent findings.

Cardiac and vascular interactions are interlinked by ventriculo-arterial coupling (VAC) [24]. Increased arterial stiffness and wave reflections elevate cardiac afterload, whereas training-induced increases in stroke volume and pulsatile bloodflow influence shear stress and endothelial signaling [25,26,27]. This bidirectional relationship makes it challenging to make causal inferences, necessitating comprehensive, vascular-focused assessment integrating vascular mechanics, circulating mediators and training exposure.

The vascular endothelium constitutes the central interface of exercise-induced vascular adaptation, maintaining homeostasis through vasoactive mediators, mainly nitric oxide (NO) and endothelin-1 (ET-1) [28]. Endothelial NO synthase (eNOS) continuously produces NO, sustaining vasodilator tone and arterial compliance. Exercise enhances both eNOS activity and NO bioavailability [29]. In addition, vasodilatory prostaglandins contribute to exercise-related vasodilation and modulate vascular reactivity, acting in concert with NO and partly counterbalancing ET-1-mediated constriction [30]. Impaired NO signaling marks endothelial dysfunction and precedes hypertension and atherosclerosis [31,32]. Conversely, ET-1 is a potent vasoconstrictor and pro-hypertrophic peptide implicated in pathological vascular remodeling [33,34]. Thus, the dynamic NO-ET-1 balance is a key determinant of vascular phenotype and may represent a mechanistic link between habitual activity and arterial structure [35,36].

Beyond endothelial mediators, endocrine-metabolic signals also shape the vascular phenotype during adolescence [37,38]. Leptin, which is secreted by adipocytes and is a key regulator of energy homeostasis, exerts context-dependent vascular effects. At physiological levels, it can activate PI3K/Akt-dependent NOS signaling, promoting vasodilation [39,40]. Chronic hyperleptinemia, by contrast, fosters OXS, sympathetic activation, and pro-atherogenic signaling [41,42]. In adolescents, elevated leptin correlates with reduced arterial distensibility independent of its role as an adiposity proxy, underscoring its direct vascular relevance [43]. Physical activity and higher fitness are linked to lower leptin and exercise interventions reduce leptin alongside improvements in body composition [44,45]. In this sense, leptin concentrations in athletic youth are generally lower, reflecting their more favorable body composition; however, periods of low energy availability can suppress leptin to very low levels [46]. Moreover, sex-specific differences are evident, with girls generally exhibiting higher leptin concentrations than boys at comparable levels of body fat, which may modulate sex-dependent vascular responses to leptin [47].

Energy metabolism and vascular function are also coupled via thyroid hormones. Free triiodothyronine (fT3) activates PI3K/Akt signaling and enhances NO-mediated relaxation and genomic pathways remodel vascular phenotype over time [48,49]. Importantly, fT3 is sensitive to energy availability, a caloric deficit can lower T3 even within the euthyroid range, while androgens, particularly testosterone, enhance peripheral T4-to-T3 conversion, contributing to the age-related rise in fT3 seen in pubertal boys. [50]. Estrogens likewise interact with thyroid hormone transport and metabolism (e.g., by increasing thyroxine-binding globulin and influencing deiodinase activity), potentially shaping peripheral T4-to-T3 conversion and fT3 trajectories in pubertal girls [51,52]. In longitudinal pediatric cohorts, higher fT3 tracks with increased SBP [53]. In a sports-cardiology context, assessing leptin and fT3 alongside vascular mechanics may help distinguish physiological remodeling from early patterns in which the beneficial CV effects of training are blunted by interfering metabolic or endocrine factors.

Lipoproteins remain central to pediatric CV risk assessment. Dyslipidemia in childhood strongly predicts adult atherosclerosis and CV events [54,55]. Mechanistically, HDL promotes vasoprotection by stimulating eNOS via SR-BI signaling (Src/PI3K/Akt/MAPK), enhancing NO bioavailability and exerting anti-inflammatory, antithrombotic effects [56,57], whereas LDL, especially oxidized LDL, impairs NO pathways and accelerates endothelial dysfunction [58]. Adverse pediatric lipid profiles correlate with greater arterial stiffness and unfavorable vascular geometry [54,55]. Exercise typically raises HDL, with more variable effects on LDL depending on training load and diet [59,60]. Nutritional factors such as diet quality, energy availability antioxidant intake, and dietary nitrate further shape these processes [61,62,63].

Overall, exercise modulates the biomarker-hemodynamic axis as aerobic training upregulates NO signaling [64] raises HDL, lowers leptin and favors blood pressure trajectories [65,66]. Conversely, physical inactivity accelerates arterial stiffening, an early marker of hypertension and CV disease [67,68]. Integrating quantified training exposure with detailed vascular phenotyping (IMT, pulse wave velocity (PWV), carotid strain/strain rate) and biomarkers panels (NO/ET-1, leptin/fT3, HDL/LDL) allows differentiation of physiological remodeling from maladaptive patterns, informing pediatric prevention.

Current evidence supports a multifactorial framework in which PA modulates endothelial mediators, metabolic hormones and lipoproteins to shape vascular function and structure, yet the mechanisms driving remodeling in highly active children remain poorly understood. Here, we address this gap by integrating detailed vascular phenotyping with a biomarker panel in young athletes to distinguish physiological from potentially maladaptive remodeling and to clarify training-biomarker-vascular links.

To this end, we formulated four specific research questions (RQs):

**RQ1:**  *How do circulating NO, ET-1, and their ratio relate to vascular function indices?*

**RQ2:**  *Is a favorable lipid profile associated with better vascular structure and function—independent of training volume or body composition?*

**RQ3:**  *Does fT3 relate to exercise performance and vascular indices?*

**RQ4:**  *Does leptin relate to the NO-ET-1—balance and vascular function?*

## 2. Materials and Methods

### 2.1. Study Design

**Figure 1:**
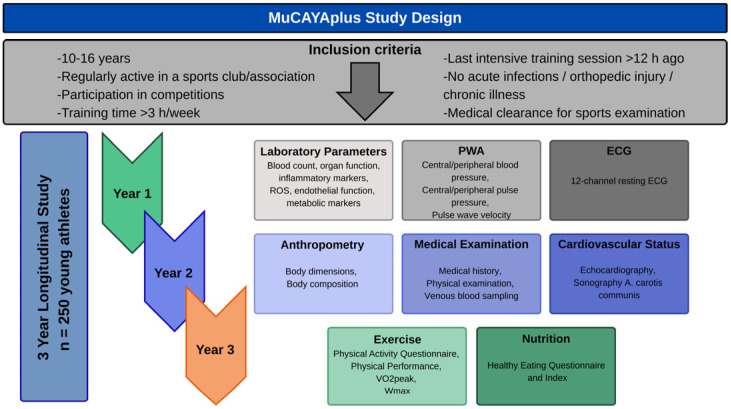
Study design overview of the MuCAYAplus study.

This analysis is based on cross-sectional data derived from the baseline cohort of the Munich Cardiovascular Adaptations in Young Athletes Plus Study (MuCAYAplus) [69], a three-year longitudinal investigation designed to examine the effects of physical activity on the cardiovascular system of young athletes. The project is conducted as a single-center study, with all assessments and data collection performed at the Institute of Preventive Pediatrics at the Technical University of Munich (TUM). Data for the present analysis was collected between November 2023 and November 2024. A detailed study protocol has been published previously [69] and is depicted in Figure 1.

### 2.2. Participants

Participants were recruited during routine sports medical examinations at the Institute’s outpatient clinic and targeted both returning patients previously examined at the institute and first-time attendees.

Participants were eligible if they regularly engaged in a primary sport and held membership in a club or association, participated in organized athletic competitions and trained at least 3 h per week. They needed documented medical clearance for cardiopulmonary exercise testing, and had to agree to attend annual follow-up examinations for three consecutive years.

Exclusion criteria included the presence of acute infection/orthopedic injury/chronic disease.

All measurements were performed ≥ 12 h after the last intensive training session to ensure adequate recovery.

Among 203 young athletes (156 boys, 47 girls), boys were significantly taller (*p* = 0.005), had greater body surface area (*p* = 0.022), more fat-free mass (*p* < 0.001), and lower body fat percentage (*p* < 0.001). They also exhibited higher SBP (*p* = 0.001), pulse wave velocity (*p* = 0.022), intima-media thickness (*p* = 0.001), and carotid diameter (*p* = 0.026). Girls had significantly lower arterial strain (*p* < 0.001) and systolic strain rate (*p* = 0.006). There were no sex differences in diastolic strain. Leptin levels were higher (*p* < 0.001), while fT3 levels were lower (*p* = 0.023) in girls. Boys had a higher peak power output and VO_2_peak (both *p* < 0.001) (Table 1).

### 2.3. Measurements

#### 2.3.1. Anthropometrics

Body weight and composition were assessed via bioelectrical impedance analysis (InBody 270 body composition analyzer, InBody Europe, Eschborn, DE). Height, waist and hip circumference were tape-measured to 0.1 cm accuracy [70] and BMI and body surface area (BSA) were calculated [71]. Age- and sex-specific z-scores for BMI, waist circumference, and hip circumference were determined using established reference values from a German pediatric cohort [72].

#### 2.3.2. Basic Cardiovascular Diagnostic

A 12-lead resting ECG (CARDIOVIT CS-200 Office system, Schiller, Baar, Switzerland) was performed in supine position. After 5 min rest, peripheral/central blood pressure and pulse wave velocity (PWV) were obtained at the brachial artery (Mobil-O-Graph device, IEM, Stolberg, Germany) and compared with age- and sex-specific references [72].

#### 2.3.3. Sonographic Measurement of Structural and Functional Vascular Parameters

All sonographic examinations were performed using the ultrasound system Canon Aplio i900 (Canon Medical Systems, Neuss, Germany). The structural vascular parameter of the Intima-Media-Thickness (IMT) of the carotid artery was measured using B-mode ultrasound in accordance with the guidelines of the Association for European Pediatric and Congenital Cardiology (AEPC).

Participants were examined in supine position with the head rotated contralateral to the side of measurement. IMT was obtained from the posterior wall of the common carotid artery, 1 cm proximal to the bifurcation, during end-diastole. Bilateral measurements were performed at standardized insonation angles (120° and 150° on the right; 210° and 240° on the left). Carotid diameter (CD)was measured inter-adventitial at the same location and phase, and the intima-diameter ratio (cIDR) was calculated as IMT/CD.

The functional properties of the vessel wall of both carotid arteries were assessed by measuring the peak circumferential strain as well as the peak systolic and diastolic strain rates using B-mode ultrasound with speckle-tracking technology. Participants were positioned supine with the head in neutral alignment and chin slightly elevated. Recordings were acquired during end-expiratory breath-hold at the proximal and distal carotid segments, reference to the maximum transverse diameter of the ipsilateral thyroid lobe and the carotid bifurcation.

#### 2.3.4. Cardiopulmonary Fitness

Cardiopulmonary exercise testing (CPET) was performed using a cycle ergometer (Excalibur, Lode B.V., Groningen, Netherlands) with a modified Godfrey protocol [73]. Participants commenced with a 2-min seated rest, after which exercise was initiated at a workload of 0.5 W/kg body mass. Ramp increments were individually adjusted to achieve exhaustion within 6–12 min, according to estimated aerobic capacity [74]. Maximal effort was defined as a respiratory exchange ratio (RER) ≥ 1.10. To ensure comparability across individuals, measures of cardiopulmonary performance were normalized to body mass (VO_2_peak in mL/min/kg; peak power output in W/kg). During testing, participants were continuously monitored via 12-lead ECG, blood pressure was recorded at 2-min intervals and oxygen saturation was intermittently assessed by pulse oximetry. Breath by breath gas exchange was measured and analyzed using the MetaMax 3B system (Cortex Biophysik, Leipzig, Germany). Following maximal exertion, all participants completed a supervised 5-min active recovery phase.

#### 2.3.5. Venous Blood Sampling and Analysis of Laboratory Parameters

Prior to exercise testing, fasting venous blood samples were collected in 7.5 mL K3EDTA tubes (01.1605.001, SARSTEDT AG & Co. KG, Nümbrecht, Germany), centrifuged at 1000g for 15 min at 6 °C, and plasma aliquots stored at −80 °C until analysis. Assessed parameters included endothelial markers (NO, ET-1), metabolic markers (leptin, fT3) and lipoproteins (LDL, HDL). Analyses of fT3, LDL and HDL were performed externally (synlab MVZ Labor, Munich, Germany). NO, ET-1 and leptin were analyzed in-house using commercially available kits according to manufacturer’s protocols.

Plasma NO metabolites (NO_2_^−^/NO_3_^−^ as approximation of NO, referred to as NO in the results/discussion) concentrations were determined after filtration (UFC501096, Merck Millipore, Merck KGaA, Darmstadt, Germany) using the Total Nitric Oxide and Nitrate/Nitrite Assay (KGE001, R&D Systems, Inc. Minneapolis, MN, USA), based on enzymatic nitrate-to-nitrite conversion and colorimetric detection (540 nm with 690 nm correction) on a Tecan Infinite M Nano+ plate reader (30190087,Tecan Group Ltd., Männedorf, Switzerland). ET-1 (DET100, R&D Systems, Inc., Minneapolis, MN, USA) and leptin (E077, Mediagnost GmbH, Reutlingen, Germany) were quantified via sandwich-ELISA, with absorbance measured at 450 nm (540 nm correction for ET-1; 590 nm for leptin).

#### 2.3.6. Physical Activity Questionnaire (MoMo-PAQ)

Physical activity was evaluated using the validated MoMo-PAQ questionnaire [75,76], capturing sports participation, leisure activity, and daily activity. Training history, discipline, weekly hours, and competition participation were recorded. Training loads were standardized as metabolic equivalent of task hours (MET-h) per week, accounting for both intensity and duration.

### 2.4. Ethical Considerations

The study has received approval from the TUM ethics committee (Project number: 516547656). All participants were provided with a detailed written information sheet about the study and were verbally informed as well. Depending on their age, written consent to participate was obtained from both the participants themselves and their legal guardians.

### 2.5. Statistical Analysis

All analyses were conducted using IBM SPSS Statistics 29 (IBM Corp., Armonk, NY, USA). A two-sided α = 0.05 was applied, and 95% confidence intervals (CIs) are reported. Continuous variables were inspected for normality via histogram, Q-Q plots, and Shapiro–Wilk tests. Non-normally distributed biomarkers (NO, ET-1, Leptin) were log-transformed to approximate normality. Descriptive statistics are presented as mean ± SD (or median [IQR]) for continuous variables. Between-group differences were examined using independent sample *t*-test or Mann–Whitney U-tests (continuous) and χ^2^-tests (categorical). Pairwise relationships between biomarkers, vascular indices and training and performance variables (MET-h/week, VO_2_peak, Watt_max_) were assessed using Pearson’s or Spearman’s correlation as appropriate.

Regression Modeling: Associations between biomarkers and vascular outcomes were examined using multivariable linear regression. Separate models were fitted for: Endothelial markers (NO, ET-1, NO/ET-1-ratio), lipid parameters (HDL, LDL, LDL/HDL-ratio), hormonal markers (fT3, leptin). Dependent variables were vascular structure and function indices: carotid strain, strain rates (systolic, diastolic), IMT, CD, cIDR and PWV. Regression coefficients β, 95%-CIs, *p*-values and adjusted R^2^ are reported. Model assumptions (linearity, homoscedasticity, multicollinearity via VIF < 5) were verified.

Models were hierarchically adjusted as follows:

Model 1: unadjusted (bivariate),
Model 2: + age, sex,
Model 3: + body composition (BSA, body fat %),
Model 4: + training volume (MET-h/week) and hemodynamic covariates (bSBP, where relevant)

Mediation and moderation analyses: Formal mediation analyses (PROCESS v4.3 macro; Model 4) tested indirect effects of candidate mediators on the relationships between NO, ET-1, leptin and fT3 and vascular outcomes. Indirect effects were estimated using bias-correlated bootstrapping with 5000 resamples and 95% bootstrapped CIs. Moderation by sex or training level level (leptin × activity; leptin × sex) was examined by including interaction terms (mean-centered variables).

Sex-stratified and matched analyses: Given significant sex differences in vascular geometry and biomarkers, primary models were rerun stratified by sex. Additionally, age-and sex-matched subsamples (1:1 nearest-neighbor matching, tolerance = 0.2 SD) were analyzed to verify robustness of observed associations.

## 3. Results

### 3.1. NO, ET-1 and Endothelial Function (RQ1)

Training volume was not related to higher NO or lower ET-1 across linear and mediation analyses. Instead, ET-1 declined modestly with age. Neither NO nor ET-1 showed robust associations with carotid deformation (strain or strain rates), and there was no evidence that training influenced vascular function indirectly via these endothelial markers. For arterial stiffness, NO, ET-1, and their ratio were unrelated to PWV; stiffness was explained chiefly by SBP and, to a lesser extent, by VO_2_peak, with a supplementary positive association for leptin in mediation frameworks. Overall, anthropometry and hemodynamics, not habitual training or endothelial biomarkers, accounted for most of the variance in vascular outcomes (Supplemental Material, Table S1).

### 3.2. Lipoprotein Profile and Vascular Structure (RQ2)

Across bivariate and multivariable analyses, circulating LDL, HDL, and their ratio showed no independent associations with carotid wall thickness, lumen size, the diameter-to-IMT ratio (cIDR), or deformation indices once core covariates were included. Carotid geometry was instead shaped predominantly by body size—diameter scaled with body surface area—and IMT was consistently lower at higher fT3 levels. Body fat contributed specifically to less favorable cIDR, while strain and strain-rate outcomes were explained mainly by sex, body fat percentage, and blood pressure rather than by lipids. Mediation analyses provided no credible indirect pathways through lipid intermediates (Supplemental Material, Table S2).

### 3.3. fT3 as a Marker of Energy Metabolism (RQ3)

After adjusting for age, sex, activity and fat-free mass, which dominated performance variance, fT3 did not account for significant variance in aerobic performance (VO_2_peak or maximal power). fT3 was also unrelated to carotid deformation (strain and strain-rate). In contrast, higher fT3 was robustly and independently linked to lower IMT, and this relationship persisted after accounting for body fat percentage; male sex was associated with thicker walls. Mediation via endothelial markers or lipids was not supported, indicating a direct structural association rather than an effect transmitted through NO/ET-1 or the lipid profile (Supplemental Material, Table S3).

### 3.4. Leptin at the Nexus of Energy Balance, and Vascular Function (RQ4)

Leptin closely tracked body fat percentage (BF%: r = 0.73; *p* < 0.001). While the bivariate link to NO was null, multivariable models (adjusted for age, sex, BF%, MET-h) showed a positive independent association between leptin and NO (B = 0.12; 95% CI 0.03–0.22; *p* = 0.015), alongside an inverse association of BF% with NO (B = −0.010; *p* = 0.005). Mediation analyses indicated a small negative indirect path via ET-1 (indirect effect = −0.017; 95% CI −0.040 to −0.001), with no credible paths through HDL/LDL and no overall indirect effect.

Leptin showed no independent association with carotid strain or systolic strain rate. In contrast, leptin was robustly and inversely related to diastolic strain rate (SRdia: B = −0.32; 95% CI −0.50 to −0.14; *p* < 0.001); notably, BF% related positively to SRdia (B = +0.022; *p* < 0.001). In multiple-mediator frameworks (ET-1, NO, HDL, LDL), the direct leptin effect on SRdia persisted (B = −0.355; *p* = 0.0002).

Regarding arterial stiffness and structure, PWV was driven by SBP and age (SBP: B = +0.029 per mmHg; *p* < 0.001; age: B = +0.054 per year; *p* = 0.0002), with no independent effect of leptin (B = 0.13; *p* = 0.115). IMT was likewise unrelated to leptin—directly or via NO/ET-1/HDL/LDL (all *p* ≥ 0.11). Collectively, leptin emerged as a signal of greater NO bioavailability and more favorable diastolic wall mechanics independent of body composition or training, whereas arterial stiffness and wall thickness remain anchored to hemodynamic load and age (Table 2).

Because sex contributed meaningfully to variance in several outcomes—most notably in carotid geometry and IMT, and alongside anthropometry and hemodynamics—and given the largely null or covariate-dependent associations for training exposure, lipids, and fT3, we next conducted sex-stratified analyses in the full cohort and replicated models in age- and sex-matched subsamples. These analyses specifically tested whether the observed relationships—particularly the leptin–NO/SRdia links and the inverse fT3–IMT association—differ by sex, to clarify potential sex-specific vascular adaptations in athletic youth.

In male participants, aerobic fitness was the dominant correlate of carotid mechanics: higher VO_2_peak aligned with higher strain (B ≈ +2.9; *p* ≤ 0.002) and SRsys (B ≈ +0.24; *p* ≈ 0.005) and lower SRdia (B ≈ −0.23; *p* ≈ 0.010). Effects persisted in matched subsamples and were independent of NO, ET-1, and NO/ET-1 (all non-significant). In female participants, VO_2_peak –deformation (strain, strain rate) links were weak or absent. PWV was chiefly explained by SBP in both sexes (≈+0.027–0.032 m/s per mmHg; *p* < 0.001); a single ET-1–PWV signal in matched males (B ≈ −0.70; *p* = 0.030) did not generalize (Supplemental Material, Table S4).

LDL, HDL, and LDL/HDL did not independently predict IMT, diameter, cIDR, or deformation in either sex. Carotid diameter scaled with BSA in both sexes, more steeply in girls (~+1.0 mm per m^2^ vs. ~+0.6–0.8 mm per m^2^ in boys). IMT showed no lipid effects; in matched girls, older age related to lower IMT (*p* ≈ 0.03–0.05). For cIDR, explained variance was low (R^2^ ≤ 0.20) and lipids were null; girls showed only a weak negative BF% trend (*p* ≈ 0.05–0.07). PWV models had high explanatory power (R^2^ ≈ 0.62–0.69) dominated by SBP (*p* < 0.001). Small inverse lipid–PWV signals in unmatched boys (e.g., LDL B ≈ −0.002; *p* ≈ 0.027) vanished after matching (Supplemental Material, Table S5).

VO_2_peak and maximal power were dominated by fat-free mass (*p* < 0.001), plus age in males and training volume in females. fT3 was unrelated to strain/strain rates and to PWV (SBP again +0.027–0.032 m/s per mmHg; *p* < 0.001), but was consistently inversely associated with IMT: unmatched males B = −0.017 (*p* = 0.015), females B = −0.044 (*p* = 0.018); after matching, females B = −0.041 (*p* = 0.038), males B = −0.025 (*p* = 0.092) (Supplemental Material, Table S6).

Leptin showed limited associations with vascular parameters in females. In males, leptin was inversely related to SRdia in the unmatched cohort (B ≈ −0.42; *p* < 0.001), an effect that was weakened after matching (B ≈ −0.23; *p* ≈ 0.25); links between leptin and strain, SRsys, PWV, and IMT were otherwise null. For NO, leptin was non-significant in both sexes (males *p* ≈ 0.07–0.09; females *p* ≈ 0.08); in unmatched males, BF% related inversely to NO (B ≈ −0.009; *p* = 0.020), attenuating with matching. PWV remained blood-pressure–driven (SBP ≈ +0.028–0.034 m/s per mmHg; *p* < 0.001), with an additional age effect in males (*p* ≤ 0.004) (Supplemental Material, Table S7).

**Figure 2:**
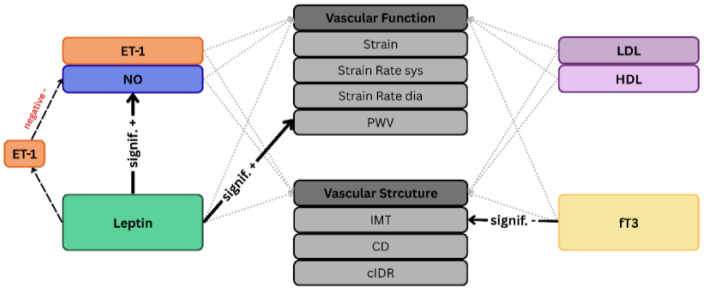
Overview of the effects of biomarkers on parameters of vascular structure and function. Grey dotted arrows imply non-significant effects, black arrows imply significant effects (+, positive; − negative), black dotted lines imply mediation effects.

An overview of the effects and mediation of all biomarkers on vascular structure and function is summarized in Figure 2.

## 4. Discussion

The present study provides novel insights into the complex determinants of vascular health in a large cohort of physically active youth. Although traditional CV risk factors such as LDL cholesterol were not significantly associated with arterial stiffness, structure, or compliance, metabolic and hormonal regulators—including free triiodothyronine (fT3) and leptin—emerged as key correlates of vascular phenotype. These findings reinforce a multifactorial model in which CV adaptations in young athletes are shaped by the interplay of hemodynamic load, endocrine signals, and body composition rather than isolated factors such as training volume or classical lipid profiles.

Leptin emerged as a salient determinant of vascular function, displaying dual vascular effects. Higher concentrations of leptin predicted increased NO concentrations, a response consistent with leptin-induced eNOS activation via the PI3K/Akt pathway [40,77]. This effect may represent a compensatory mechanism counterbalancing leptin’s pro-hypertensive action via sympathetic activation [78,79]. In contrast, leptin showed no independent association with PWV, which was almost entirely explained by SBP and age. This dissociation, leptin linked to local carotid wall mechanics but not to global macrovascular stiffness, suggests that in early adolescence, central arterial stiffness may appear unaffected by leptin, while subtle leptin-related differences are already detectable at the level of local wall motion. Carotid strain rate may thus be a sensitive early marker of adipokine-related vascular effects, highlighting the need for longitudinal follow up to determine whether these functional alterations precede later macrovascular stiffening. Our data also indicate that physiological leptin levels in non-adipose youth are vascularly active and, under healthy conditions, may exert context-dependent, potentially protective effects. Mediation analyses indicated a small negative indirect path via ET-1, implying that ET-1 partially attenuates leptin’s NO-enhancing influence. Simultaneously, higher leptin concentrations were associated with lower SRdia, which in this cohort reflects more favorable wall relaxation. This suggests that leptin may modulate vasodilatory tone and diastolic mechanics by acting on eNOS pathway, although chronic elevations could still contribute to vascular stress under different metabolic conditions [79].

Interestingly, leptin’s vascular associations were evident only in male participants. In boys, leptin correlated positively with NO and negatively with SRdia, suggesting functional vascular sensitivity. In contrast, despite exhibiting higher leptin concentrations per se [37,80], no such relationship was observed. This striking sex difference may reflect divergent leptin sensitivity, as female vascular tissue shows estrogen-mediated NO responsiveness and relative leptin resistance, while in males, leptin exerts stronger sympathetic and direct endothelial effects [81,82].

FT3 emerged as a robust hormonal correlate of vascular structure. Higher fT3 was independently associated with thinner carotid IMT, consistent with a potential protective role for thyroid hormones in arterial remodeling [83]. This association remained significant after adjusting for body composition and was not mediated by blood lipids or endothelial biomarkers, suggesting direct vascular actions of fT3. Mechanistically, fT3 promotes vasodilation through eNOS activation and upregulates LDL receptor expression, enhancing lipid clearance [49,84,85]. Hypothyroidism is known to promote atherosclerosis via endothelial dysfunction and dyslipidemia [86]. Our findings extend this paradigm to healthy adolescents, revealing that even within the normal range, fT3 variability may shape vascular phenotype, in which even relatively small deviations within the physiological range could already be vascularly relevant. The fT3–IMT relationship was particularly pronounced in females, aligning with studies suggesting increased vascular sensitivity to thyroid status in women [87]. This is relevant in athletic girls, where energy deficiency may suppress thyroid function [88]. Subtle reductions in fT3, even within the euthyroid range, could indicate low energy availability and contribute to early vascular remodeling, raising the possibility that fT3 may serve as an early subclinical marker, and candidate biomarker for clinical screening, of vascular change in youth. Collectively, the sex-specific associations observed for fT3 and leptin highlight that endocrinologic-vascular interactions during adolescence are context-dependent and likely modulated my maturational and hormonal status. These findings emphasize the importance of sex-differentiated CV profiling in youth athletes [89].

Habitual training volume was not significantly associated with circulating NO or ET-1, diverging from adult studies in which training increases NO and lowers ET-1 [90,91,92]. This discrepancy likely reflects a physiological ceiling effect in our cohort, as adolescents engaged in consistent activity may already exhibit optimal endothelial function. Supporting this, prior pediatric studies report training-induced NO increases primarily in obese or sedentary youth, with minimal changes in healthy, active populations [93]. ET-1 declined modestly with age, consistent with findings in adult athletes and potentially indicative of cumulative training or maturational influences [94]. Endocrine modulators such as thyroid hormones and leptin may further regulate these endothelial pathways, linking systemic metabolism with vascular homeostasis. These interactions could represent a key interface between metabolic adaptation and vascular function in young athletes [95].

Importantly, neither NO nor ET-1 correlated with carotid strain indices or pulse wave velocity (PWV). These results suggest that inter-individual differences in resting endothelial biomarkers may not translate directly into measurable differences in arterial elasticity in this age group. Instead, hemodynamic factors, especially SBP, played a dominant role. SBP was the strongest independent predictor of PWV, underscoring the influence of mechanical load on arterial stiffness even in youth [96]. No mediation by NO or ET-1 was observed, implying that other mechanisms—such as vascular smooth muscle tone or autonomic regulation—may drive training-induced vascular adaptation.

Aerobic fitness, as measured by VO_2_peak, was associated with increased carotid elasticity in males but not females. This sex difference could reflect variations in vascular responsiveness or hormonal buffering. Female participants exhibited greater arterial compliance overall, independent of fitness, possibly due to estrogen-mediated endothelial protection or inherently lower arterial load.

Lipid profiles (LDL, HDL, and LDL/HDL ratio) were largely inert with respect to vascular outcomes. There were no associations between lipid levels and carotid intima-media thickness (IMT), arterial diameter, or functional indices such as strain. These null findings likely reflect both the favorable lipid profiles of the cohort and limited cumulative exposure time. In contrast to adult data linking LDL elevations with increased IMT [97], vascular effects of lipids may require prolonged dyslipidemia, which is rare in this population. Similarly, prior studies in pediatric populations show lipid improvements with exercise primarily in obese or dyslipidemic children, with minimal effects in normolipidemic youth [98,99].

Body size was a significant determinant of carotid geometry, with arterial diameter scaling with body surface area. Functional vascular indices were strongly influenced by body fat, SBP, and sex. Higher body fat percentage predicted reduced strain and strain rate, consistent with early vascular stiffening, potentially via inflammatory or mechanical pathways [100,101]. These associations were independent of lipid levels, again emphasizing non-lipid metabolic influences on vascular function.

### Limitations

Although the study has key strengths, several limitations need to be acknowledged. First, the cross-sectional design inherently limits causal inference. While we observed associations between leptin, fT3, and vascular parameters, temporal relationships remain unknown. The planned longitudinal follow-up within the MuCAYAplus study [69] will be essential to clarify whether these biomarkers predict subsequent vascular remodeling or primarily reflect concurrent adaptation. Second, although the overall cohort was relatively large (*n* = 203), sex distribution was imbalanced with nearly three times as many male participants, limiting power for female-specific analyses. Pubertal status was not directly assessed, so inter-individual differences in maturation, strong determinants for both vascular and hormonal profiles, may not be fully captured despite adjustment for age and sex. Future work should include Tanner staging or pubertal hormone markers.

Third, training exposure was derived from self-reported MoMo-PAQ data and converted to MET-hours/week. Even with a validated instrument, recall and social desirability bias are possible, particularly in younger participants. Objective monitoring (e.g., accelerometry, wearables, heart rate) would improve does-response estimation. Fourth, we focused on a targeted biomarker panel (NO, ET-1, leptin, fT3, HDL, LDL), which does not encompass the full complexity of vascular signaling. Inflammatory, OXS and insulin sensitivity markers, endothelial progenitor cells, as well as nutritional status and dietary intake—all relevant for adolescent vascular adaptation, especially under conditions of high training load or energy imbalance—were not assessed and should be integrated in future studies. Fifth, vascular outcomes were confined to carotid measurements, which, although robust and clinically meaningful, reflect regional rather than systemic arterial properties. Central arterial stiffness (e.g., aortic PWV) and myocardial-vascular coupling parameters (e.g., ventricular-arterial coupling indices) would provide further insight into cardiovascular integration in youth athletes. In addition, strain analysis was based on 2D speckle tracking, which, although validated, is operator-dependent and may be sensitive to image quality and insonation angle. Replication using alternative imaging modalities (e.g., MRI-based vascular stiffness or tonometry) could strengthen conclusions. Sixth, biomarkers were assessed in the fasting resting state, which may not reflect dynamic responses to acute or chronic exercise stimuli. Some vascular adaptations may only be detectable in response to stress, which were not evaluated here. Incorporating functional vascular tests would allow for a more comprehensive assessment of endothelial responsiveness and vascular reserve. Seventh, while fT3 emerged as a strong correlate of IMT, it was assessed in isolation without measurement of upstream regulators (TSH, T4) or markers of energy availability (e.g., leptin:ghrelin ratio, caloric intake, resting energy expenditure). This limits interpretation of the fT3–vascular link, particularly in the context of potential energy deficiency in young athletes. Future studies should incorporate comprehensive thyroid panels and validated indices of energy status [102] to distinguish physiological versus pathophysiological hormone patterns. Finally, while we excluded individuals with acute illness or known chronic disease, unmeasured determinants such as genetic, socioeconomic, and environmental factors (e.g., pollution, psychosocial stress) could have contributed to inter-individual variability in vascular and/or metabolic phenotypes.

Despite these limitations, this study provides comprehensive analyses of vascular and endocrine determinants in physically active youth and lays the groundwork for longitudinal evaluation of CV adaptation during adolescence. In practical terms, these findings could argue for routine surveillance measurements for interpreting vascular indices alongside endocrine–metabolic signals, blood pressure and body composition to guide individualized training, nutrition, and follow-up.

## 5. Conclusions

Taken together, our findings support a multifactorial model in which vascular health in highly active youth is shaped by the interplay of hemodynamic load, metabolic-endocrine signals, and body composition, rather than by traditional risk factors alone. While habitual physical activity confers overall vascular protection, our data reveal that individual variation in biomarkers such as free triiodothyronine (fT3), leptin, and SBP contributes meaningfully to arterial structure and function. These effects were sex-specific, underscoring the need for differentiated CV profiling during adolescence. Although conventional lipid screening remains an important component of CV risk assessment, the lack of strong lipid–vascular associations in this healthy cohort suggests that, in active youth, integrative biomarker panels may provide additional insight into early vascular phenotypes beyond lipids alone. By integrating endocrine, metabolic and vascular data, this study provides novel evidence on how training interacts with hormonal regulation to shape vascular adaptation in youth. Future longitudinal work should clarify whether these biomarker-vascular relationships predict long-term CV remodeling and performance potential. In sum, our findings underscore that vascular health in athletic youth is not solely a function of training volume but reflects the dynamic integration of metabolic and endocrine pathways within CV development.

## Figures and Tables

**Table 1:** Study characteristics.

	*n*	Total (M ± SD)	*n*	Boys (M ± SD)	*n*	Girls (M ± SD)	*p*
Age (yrs)	203	13.5 ± 1.6	156	13.6 ± 1.6	47	13.1 ± 1.61	0.058
Body mass (kg)	203	52.5 ± 12.3	156	53.4 ± 12.6	47	49.4 ± 11.0	0.051
Body height (cm)	203	165.6 ± 12.7	156	166.8 ± 13.3	47	161.7 ± 9.7	0.005 **
BMI (kg/m^2^)	203	18.8 ± 2.3	156	18.9 ± 2.1	47	18.7 ± 2.7	0.556
BSA (m^2^)	203	1.5 ± 0.2	156	1.5 ± 0.2	47	1.5 ± 0.2	0.022 *
Body Fat (%)	200	12.9 ± 5.9	154	11.7 ± 5.8	46	16.8 ± 4.5	<0.001 ***
FFM (kg)	200	45.3 ± 11.9	154	46.8 ± 12.6	46	40.3 ± 8.0	<0.001 ***
bSBP (mmHg)	203	110.72 ± 9.37	156	111.89 ± 9.08	47	106.82 ± 9.36	0.001 ***
bDBP (mmHg)	203	62.35 ± 6.15	156	62.69 ± 5.95	47	61.25 ± 6.69	0.161
PWV (m/s)	203	4.58 ± 0.405	156	4.62 ± 0.41	47	4.46 ± 0.36	0.022 *
IMT (mm)	203	0.480 ± 0.037	156	0.48 ± 0.03	47	0.46 ± 0.03	0.001 ***
CD (mm)	203	5.97 ± 0.35	156	6.00 ± 0.36	47	5.87 ± 0.30	0.026 *
cIDR	203	0.08 ± 0.006	156	0.08 ± 0.007	47	0.08 ± 0.006	0.142
Strain (%)	203	9.89 ± 2.56	156	10.32 ± 2.55	47	8.46 ± 2.06	<0.001 ***
SRsys (m/s)	203	1.18 ± 0.26	156	1.21 ± 1.21	47	1.09 ± 0.24	0.006 **
SRdia (m/s)	203	−0.99 ± 0.29	156	−0.99 ± 0.29	47	−1.01 ± 0.26	0.735
MET-h/week	203	96.89 ± 46.07	156	96.28 ± 40.51	47	98.90 ± 61.52	0.785
NO (µmol/L)	202	23.89 ± 10.65	156	24.18 ± 9.62	46	22.88 ± 13.66	0.468
ET-1 (pg/mL)	202	1.10 ± 0.31	156	1.09 ± 0.31	46	1.11 ± 0.33	0.793
Leptin (ng/mL)	194	3.01 ± 4.62	148	2.15 ± 3.81	46	5.77 ± 5.85	<0.001 ***
HDL (mg/dL)	201	57.49 ± 10.82	154	56.74 ± 10.50	47	59.95 ± 11.57	0.075
LDL (mg/dL)	201	92.60 ± 24.83	154	91.42 ± 25.14	47	96.46 ± 23.61	0.224
fT3 (pg/mL)	201	3.74 ± 0.42	154	3.78 ± 0.41	47	3.62 ± 0.44	0.023 *
Watt_max_ (W)	203	228.15 ± 67.77	156	239.50 ± 69.35	47	190.48 ± 45.60	<0.001 ***
VO_2_peak (l/min)	203	2.57 ± 0.72	156	2.71 ± 0.73	47	2.12 ± 0.47	<0.001 ***
VO_2_peak_rel (ml/min/kg)	203	48.99 ± 6.70	156	50.66 ± 5.93	47	43.46 ± 6.17	<0.001 ***

BMI, body mass index; BSA, body surface area; FFM, fat free mass; bSBP, systolic blood pressure; bDBP, diastolic blood pressure; PWV, pulse wave velocity; IMT, intima-media thickness; CD, carotid diameter; cIDR, carotid intima-diameter-ratio; SRsys, systolic strain rate; SRdia, diastolic strain rate; MET-h, metabolic equivalent of task hours; NO, nitric oxide; ET-1, endothelin-1; HDL, high-density lipoprotein; LDL, low-density lipoprotein; fT3, free triiodothyronine; Watt_max_, maximal power output; VO_2_peak, maximal oxygen uptake. Significant results are marked * *p* < 0.05, ** *p* < 0.01, *** *p* < 0.001.

**Table 2:** Direct and indirect effects of leptin on vascular and endothelial outcomes in young athletes.

N	Outcome	Model	Direct Effect of Leptin [B, 95% CI], *p*	Indirect Effects (PROCESS, B, 95%, Boot-CI)	Signif. Covariates (b, *p*)	R^2^/adj. R^2^
192	NO	Multivariate (age, sex, BF%, MET-h)	0.112 [0.015–0.209], *p* = 0.025 *		BF% −0.010, *p* = 0.005 **	0.053/0.028
190		PROCESS (M: ET-1, HDL, LDL; +Covariates)	0.123 [0.025–0.222], *p* = 0.015 *	−0.012 [−0.045; 0.018]	ET-1-path: −0.017 [−0.040; −0.001]; HDL/LDL n. s.	0.097
192	SRdia	Multivariate (age, sex, BF%, MET-h, bSBP)	−0.322 [−0.50; −0.14], *p* < 0.001 ***		BF% +0.022, *p* < 0.001 ***	0.097/0.067
190		PROCESS (M:ET-1, HDL, LDL, NO; +Covariates)	−0.355 [−0.542; −0.168], *p* = 0.0002 ***	0.037 [−0.024; 0.101]	Single-path via ET-1/HDL/LDL/NO all n. s.	0.108
192	SRsys	Multivariate (age, sex, BF%, MET-h, bSBP)	0.099 [−0.07; 0.26], *p* = 0.244		Sex +0.125, *p* = 0.022 *; BF% −0.010, *p* = 0.084; bSBP +0.003, *p* = 0.122	0.101/0.072
190		PROCESS (M: ET-1, HDL, LDL; +Covariates)	0.139 [−0.035; 0.312], *p* = 0.117	−0.046 [−0.120; 0.019]	bSBP +0.0044, *p* = 0.05 *; Sex +0.1125, *p* = 0.04 *; BF% −0.0124, *p* = 0.043 *; (ET-1 t = 1.79, *p* = 0.075)	0.137
192	Strain	Multivariate (age, sex, BF%, MET-h, bSBP)	1.114 [−0.38; 2.61], *p* = 0.146		Sex +1.784, *p* < 0.001 ***; BF% −0.125, *p* = 0.018 *; Alter +0.260, *p* = 0.034 *	0.183/0.161
190		PROCESS (M: ET-1, HDL, LDL; +Covariates)	1.565 [−0.003; 3.133], *p* = 0.0504	−0.382 [−1.077; 0.225]	ET-1 +3.290, *p* = 0.034 *; Sex +1.645, *p* = 0.001 ***; BF% −0.153, *p* = 0.0061 ***; bSBP +0.038, *p* = 0.064	0.231
192	PWV	Multivariate (age, sex, BF%, MET-h, bSBP)	0.143 [−0.02; 0.30], *p* = 0.075		bSBP +0.031, *p* < 0.001 ***; Alter +0.054/J, *p* = 0.0002 ***	0.637/0.625
190		PROCESS (M: ET-1, HDL, LDL; +Covariates)	0.131 [−0.032; 0.294], *p* = 0.115	0.003 [−0.058; 0.064]	LDL −0.0018, *p* = 0.0206; others n. s.	0.650
192	IMT	Multivariate (age, sex, BF%, MET-h, bSBP)	−0.002 [−0.026; 0.022], *p* = 0.839			0.119/0.091
190		PROCESS (M: ET-1, HDL, LDL; +Covariates)	−0.001 [−0.025; 0.023], *p* = 0.939	−0.0018 [−0.0097; 0.0055]		0.137

NO, nitric oxide; ET-1, endothelin-1; HDL, high-density lipoprotein; LDL, low-density lipoprotein; SRsys, systolic strain rate; SRdia, diastolic strain rate; PWV, pulse wave velocity; IMT, intima-media thickness; BF%, body fat percentage; MET-h, metabolic equivalent of task hours; MET, metabolic equivalent of task. Significant results are marked * *p* < 0.05, ** *p* < 0.01, *** *p* < 0.001.

## Data Availability

The datasets presented in this article are not readily available due to privacy and ethical restrictions. The datasets are derived from a pediatric study, and therefore contain sensitive personal and health-related information. Requests to access the datasets should be directed to the corresponding author.

## Supplementary Materials

The following supporting information can be downloaded at: https://www.mdpi.com/article/10.3390/biom15121726/s1, Table S1: Direct and indirect effects of NO, ET-1 and the NO-ET-1-ratio on vascular outcomes in young athletes; Table S2: Direct and indirect effects of LDL, HDL and the LDL-HDL-ratio on functional and structural vascular outcomes in young athletes; Table S3: Direct and indirect effects of fT3 on exercise performance and functional and structural vascular outcomes in young athletes; Table S4: Sex-specific differences in direct effects of NO, ET-1 and the NO-ET-1-ratio on functional and structural vascular outcomes in young athletes and an age-and sex-matched cohort; Table S5: Sex-specific differences in direct effects of LDL, HDL and the LDL-HDL-ratio on functional and structural vascular outcomes in young athletes and an age-and sex-matched cohort; Table S6: Sex-specific differences in direct effects of fT3 on exercise performance and functional and structural vascular outcomes in young athletes and an age-and sex-matched cohort; Table S7: Sex-specific differences in direct effects of leptin on endothelial and functional and structural vascular outcomes in young athletes and an age-and sex-matched cohort.

## Abbreviations

The following abbreviations are used in this manuscript:

CV	Cardiovascular
OXS	Oxidative Stress
PA	Physical Activity
IMT	Intima-Media Thickness
VAC	Ventriculo-Arterial Coupling
NO	Nitric Oxide
ET-1	Endothelin-1
eNOS	Endothelial NO Synthase
PI3K	Phosphatidylinositol 3-kinase
Akt	Protein Kinase B
fT3	Free Triiodothyronine
BMI	Body Mass Index
BF%	Body Fat Percentage
HDL	High Density Lipoprotein
SR-BI	Scavenger Receptor class B type 1
Src	Tyrosine Protein Kinase Src
MAPK	Mitogen-Activated Protein Kinase
LDL	Low Density Lipoprotein
PWV	Pulse Wave Velocity
MuCAYA	Munich Cardiovascular Adaptation in Young Athletes
