# Supplementary material for: Molecular Drivers of Vascular Adaptation in Young Athletes: An Integrative Analysis of Endothelial, Metabolic and Lipoprotein Biomarkers
Source: Biomolecules. 2025 Dec 11;15(12):1726. doi: 10.3390/biom15121726 (PMC12730266; doi:10.3390/biom15121726)
Supplement: Supplementary file 1 [file biomolecules-15-01726-s001.zip › biomolecules-4033975-supplementary.pdf]

Supplemental Material

Table S1: Direct and indirect effects of NO, ET-1 and the NO-ET-1-ratio on vascular outcomes in young athletes.

N	Outcome	Model	Direct effect (B, 95% CI), p	Indirect effects (PROCESS, B, 95%, Boot-CI)	Signif. Covariates (b, p)	R^2/adj. R^2
201	Strain	Multivariate (NO, age, sex, bSBP, VO_2peak, BF%, MET-h)	−0.01 [−2.02, 2.00], p=0.992		VO_2peak +1.94, p<0.001***; Sex +0.89, p=0.034*	0.256 / 0.233
202		Multivariate (ET1, age, sex, bSBP, VO_2peak, BF%, MET-h)	2.33 [−0.32, 4.99], p=0.087		VO_2peak +1.93, p<0.001***; Sex +0.87, p=0.036*	0.267 / 0.245
202		Multivariate (NO/ET1, age, sex, bSBP, VO_2peak, BF%, MET-h)	−0.95 [−2.65, 0.74], p=0.273		VO_2peak +1.94, p<0.001***; Sex +0.92, p=0.028*	0.261 / 0.238
192		PROCESS (M: ET-1, leptin, fT3; HDL; LDL +Covariates)	0.16 [−1.99, 2.32], p=0.883	0.37 [−0.32, 1.09]	VO_2peak +2.23, p<0.001***	0.280
192		PROCESS (M: NO, leptin, fT3 HDL; LDL +Covariates)	2.37 [−0.46, 5.20], p=0.101	0.16 [−0.52, 0.95]	VO_2peak +2.23, p<0.001***; Age −0.36, p=0.043*	0.277
192		PROCESS (M: leptin, fT3; HDL; LDL +Covariates)	−0.74 [−2.53, 1.05], p=0.419	0.11 [−0.33, 0.56]	VO_2peak +2.22, p<0.001***	0.268
202	SRsys	Multivariate (NO, age, sex, bSBP, VO_2peak, BF%, MET-h)	−0.06 [−0.28, 0.17], p=0.627		VO_2peak +0.13, p=0.004**	0.120 / 0.093
202		Multivariate (ET1, age, sex, bSBP, VO_2peak, BF%, MET-h)	0.21 [−0.10, 0.51], p=0.184		VO_2peak +0.13, p=0.004**	0.127 / 0.100
202		Multivariate (NO/ET1, age, sex, bSBP, VO_2peak, BF%, MET-h)	−0.12 [−0.32, 0.07], p=0.209		VO_2peak +0.13, p=0.004**	0.126 / 0.099
192		PROCESS (M: ET-1, leptin, fT3; HDL; LDL +Covariates)	−0.07 [−0.32, 0.18], p=0.575	0.06 [−0.01, 0.15]	VO_2peak +0.15, p=0.0015**	0.149

192		PROCESS (M: NO, leptin, fT3 HDL; LDL +Covariates)	0.22 [−0.10, 0.54], p=0.180	0.00 [−0.08, 0.09]	VO$_2$peak +0.15, p=0.0015**	0.149
192		PROCESS (M: leptin, fT3; HDL; LDL +Covariates)	−0.12 [−0.33, 0.08], p=0.234	0.03 [−0.02, 0.08]	VO$_2$peak +0.15, p=0.0015**	0.146
202	**SRdia**	Multivariate (NO, age, sex, bSBP, VO$_2$peak, BF%, MET-h)	−0.00 [−0.26, 0.26], p=0.988		–	0.045 / 0.015
202		Multivariate (ET1, age, sex, bSBP, VO$_2$peak, BF%, MET-h)	−0.19 [−0.53, 0.16], p=0.285		–	0.050 / 0.021
202		Multivariate (NO/ET1, age, sex, bSBP, VO$_2$peak, BF%, MET-h)	0.07 [−0.14, 0.29], p=0.505		–	0.047 / 0.018
192		PROCESS (M: ET-1, leptin, fT3; HDL; LDL +Covariates)	0.07 [−0.21, 0.34], p=0.633	−0.06 [−0.16, 0.04]	Leptin −0.15, p=0.009**; VO$_2$peak −0.13, p=0.013*	0.095
192		PROCESS (M: NO, leptin, fT3 HDL; LDL +Covariates)	−0.18 [−0.53, 0.17], p=0.315	0.03 [−0.09, 0.16]	Leptin −0.15, p=0.009**; VO$_2$peak −0.13, p=0.013*	0.095
192		PROCESS (M: leptin, fT3; HDL; LDL +Covariates)	0.11 [−0.12, 0.34], p=0.349	−0.04 [−0.12, 0.05]	Leptin −0.14, p=0.010*; VO$_2$peak −0.13, p=0.014*	0.094
202	**PWV**	Multivariate (NO, age, sex, bSBP, VO$_2$peak, BF%, MET-h)	0.09 [−0.14, 0.31], p=0.448		bSBP +0.029, p<0.001***; VO$_2$peak +0.136, p=0.002**	0.637 / 0.626
202		Multivariate (ET1, age, sex, bSBP, VO$_2$peak, BF%, MET-h)	−0.06 [−0.36, 0.24], p=0.684		bSBP +0.029, p<0.001***; VO$_2$peak +0.136, p=0.002**	0.636 / 0.625
202		Multivariate (NO/ET1, age, sex, bSBP, VO$_2$peak, BF%, MET-h)	0.09 [−0.10, 0.28], p=0.366		bSBP +0.029, p<0.001***; VO$_2$peak +0.136, p=0.002**	0.638 / 0.626
192		PROCESS (M: ET-1, leptin, fT3; HDL; LDL +Covariates)	0.10 [−0.13, 0.33], p=0.376	−0.05 [−0.18, 0.04]	bSBP +0.030, p<0.001***; VO$_2$peak +0.146, p=0.001***; Leptin +0.136, p=0.004**	0.668

192		PROCESS (M: NO, leptin, fT3 HDL; LDL +Covariates)	0.01 [−0.29, 0.31], p=0.966	−0.05 [−0.17, 0.05]	bSBP +0.030, p<0.001***; VO_2peak +0.146, p=0.001***; Leptin +0.138, p=0.004**	0.668
192		PROCESS (M: leptin, fT3; HDL; LDL +Covariates)	0.06 [−0.13, 0.25], p=0.506	−0.01 [−0.09, 0.05]	bSBP +0.030, p<0.001***; VO_2peak +0.146, p=0.001***; Leptin +0.136, p=0.004**	0.668

*NO, nitric oxide; ET-1, endothelin-1; HDL, high-density lipoprotein; LDL, low-density lipoprotein; SRsys, systolic strain rate; SRdia, diastolic strain rate; PWV, pulse wave velocity; IMT, intima-media thickness; bSBP, systolic blood pressure; BF%, body fat percentage; FFM, fat free mass; MET-h, metabolic equivalent of task hours; BMI, body mass index; BSA, body surface area. Significant results are marked *p<0.05, **p<0.01, ***p<0.001.*

Table S2: Direct and indirect effects of LDL, HDL and the LDL-HDL-ratio on functional and structural vascular outcomes in young athletes.

**N**	**Outcome**	**Model**	**Direct effect B, [95% CI), p**	**Indirect effects (PROCESS, B, 95%, Boot-CI)**	**Signif. Covariates (b, p)**	**R^2/adj. R^2**
198	**IMT**	Multivariate (HDL, age, sex, bSBP, BSA, BF%, MET-h)	0.000 [—], p=0.685		BSA 0.086, p<0.001***; Age −0.006, p=0.015*	0.232 / 0.204
198		Multivariate (LDL, age, sex, bSBP, BSA, BF%, MET-h)	−0.00005 [—], p=0.654		BSA 0.084, p<0.001***; Age −0.006, p=0.014*	0.233 / 0.204
198		Multivariate (LDL/HDL, age, sex, bSBP, BSA, BF%, MET-h)	0.000 [—], p=0.696		Age −0.006, p=0.014*; BSA +0.085, p<0.001***	0.232 / 0.204
190		PROCESS (M: ET-1, NO, leptin, fT3 LDL; +Covariates)	−0.0001 [−0.0006; 0.0004], p=0.747	−0.0001 [−0.0003; 0.0001]	fT3 −0.020, p=0.003**; Sex 0.0147, p=0.048*	0.178
190		PROCESS (M: ET-1, NO, leptin, fT3 HDL; +Covariates)	−0.0002 [−0.0004; 0.0001], p=0.151	0.0000 [−0.0001; 0.0001]	fT3 −0.020, p=0.003**; Sex 0.0147, p=0.048*	0.178
190		PROCESS (M: ET-1, NO, leptin, fT3; +Covariates)				
198	**CD**	Multivariate (HDL, age, sex, bSBP, BSA, BF%, MET-h)	0.000 [—], p=0.861		BSA +0.695, p<0.001***	0.189 / 0.159
198		Multivariate (LDL, age, sex,	B=0.000 [—], p=0.654		BSA +0.678, p<0.001***	0.190 / 0. 160

		bSBP, BSA, BF%, MET-h)				
198		Multivariate (LDL/HDL, age, sex, bSBP, BSA, BF%, MET-h)	0.000 [—], p=0.710		BSA +0.689, p<0.001***	0.189 / 0.159
190		PROCESS (M: ET-1, NO, leptin, fT3 LDL; +Covariates)	0.0020 [−0.0028; 0.0068], p=0.41	Total: −0.0004 [−0.0020; 0.001] — all n. s.	BSA +0.742, p<0.001***	0.215
190		PROCESS (M: ET-1, NO, leptin, fT3 HDL; +Covariates)	.0001 [−0.0018; 0.0021], p=0.913	Total: 0.0000 [−0.0004; 0.0005] — all n. s.	BSA +0.742, p<0.001***	0.215
190		PROCESS (M: ET-1, NO, leptin, fT3; +Covariates)	−.0132 [−0.0981; 0.0717], p=0.759	Total: 0.0067 [−0.0127; 0.0333] — all n. s.	BSA +0.708, p<0.001***	0.213
198	cIDR	Multivariate (HDL, age, sex, bSBP, BSA, BF%, MET-h)	0.000 [—], p=0.907		BF% 0.000; p=0.013*	0.059 / 0.025
198		Multivariate (LDL, age, sex, bSBP, BSA, BF%, MET-h)	0.000 [—], p=0.932		BF% 0.000; p=0.014*	0.059 / 0.025
198		Multivariate (LDL/HDL, age, sex, bSBP, BSA, BF%, MET-h)	0.0000 [—], p=0.978		BF% −0.000 p=0.014*	0.059 / 0.025
190		PROCESS (M: ET-1, NO, leptin, fT3 LDL; +Covariates)	0.0000 [−0.0001; 0.0001], p=0.879		No covariate p<0.05	0.113
190		PROCESS (M: ET-1, NO, leptin, fT3 HDL; +Covariates)	0.0000 [−0.0001; 0.0000], p=0.643	Total: .0000 [0.0000; 0.0000] — all n. s.	fT3 −0.0030, p=0.025*	0.113
190		PROCESS (M: ET-1, NO, leptin, fT3; +Covariates)	−0.0004 [−0.0022; 0.0014], p=0.641	0.0001 [−0.0005; 0.0007]	fT3 −0.0030, p=0.023*	0.113
198	Strain	Multivariate (HDL, age, sex, bSBP, BSA, BF%, MET-h)	−0.020 [—], p=0.238		Sex +1.228, p=0.006**	0.175 / 0.150
198		Multivariate (LDL, age, sex, bSBP, BSA, BF%, MET-h)	−0.008 [—], p=0.287		Sex +1.269, p=0.005**	0.174 / 0.148

		Multivariate (LDL/HDL, age, sex, bSBP, BSA, BF%, MET-h)	+0.074, [—], p=0.813		Sex +1.282, p=0.004**	0.170 / 0.144
190		PROCESS (M: ET-1, NO, leptin, fT3 LDL; +Covariates)	−0.0198 [−0.0542; 0.0147], p=0.259	−0.0042 [−0.0165; 0.0068]	ET1 +3.29, p=0.034*; Sex +1.64, p=0.001***; BF% −0.153, p=0.006**	0.231
190		PROCESS (M: ET-1, NO, leptin, fT3 HDL; +Covariates)	−0.0092 [−0.0234; 0.0050], p=0.203	0.0000 [−0.0044; 0.0044]	ET1 +3.29, p=0.034*; Sex +1.64, p=0.001***; BF% −0.153, p=0.006**	0.231
		PROCESS (M: ET-1, NO, leptin, fT3; +Covariates)	−0.0242 [−0.6569; 0.6086], p=0.940	+0.0862 [−0.1293; 0.3039]	ET1 +3.53, p=0.023*; Sex +1.74, p=0.0005***; BF% −0.157, p=0.005**	0.218
198	**SRsys**	Multivariate (HDL, age, sex, bSBP, BSA, BF%, MET-h)	−0.002 [—], p=0.359		No covariate p<0.05	0.088 / 0.059
198		Multivariate (LDL, age, sex, bSBP, BSA, BF%, MET-h)	−0.001 [—], p=0.399		No covariate p<0.05	0.087 / 0.058
		Multivariate (LDL/HDL, age, sex, bSBP, BSA, BF%, MET-h)	+0.005, [—], p=0.887		No covariate p<0.05	0.084 / 0.055
190		PROCESS (M: ET-1, NO, leptin, fT3 LDL; +Covariates)	−0.0019 [−0.0057; 0.0019], p=0.335	−0.0002 [−0.0014; 0.0009]	Sex +0.1125, p=0.040*; BF% −0.0124, p=0.043*; bSBP +0.0044, p=0.050*	0.137
190		PROCESS (M: ET-1, NO, leptin, fT3 HDL; +Covariates)	−0.0007 [−0.0023; 0.0008], p=0.353	0.0000 [−0.0005; 0.0004]	Sex +0.1125, p=0.040*; BF% −0.0124, p=0.043*; bSBP +0.0044, p=0.050*	0.137
		PROCESS (M: ET-1, NO, leptin, fT3; +Covariates)	+0.0014 [−0.0685; 0.0712], p=0.969	+0.0045 [−0.0161; 0.0252]	Sex +0.1205, p=0.027*; BF% −0.0127, p=0.038*; bSBP +0.0043, p=0.057	0.128
198	**SRdia**	Multivariate (HDL, age, sex, bSBP, BSA, BF%, MET-h)	+0.002 [—], p=0.235		No covariate p<0.05	0.037 / 0.007
198		Multivariate (LDL, age, sex, bSBP, BSA, BF%, MET-h)	0.000 [—], p=0.912		No covariate p<0.05	0.030 / 0.000

198		Multivariate (LDL/HDL, age, sex, BSBP, BSA, BF%, MET-h)	−0.046 [—], p=0.233		No covariate p<0.05	0.038 / 0.007
190		PROCESS (M: ET-1, NO, leptin, fT3 LDL; +Covariates)	+0.0023 [−0.0018; 0.0064], p=0.261	+0.0004 [−0.0013; 0.0024]	Leptin −0.380, p=0.0001***; BF% +0.0256, p=0.0001***; bSBP −0.0050, p=0.041*	0.135
190		PROCESS (M: ET-1, NO, leptin, fT3 HDL; +Covariates)	−0.0002 [−0.0018; 0.0015], p=0.855	0.0000 [−0.0008; 0.0008]	leptin −0.380, p=0.0001***; BF% +0.0256, p=0.0001***; bSBP −0.0050, p=0.041*	0.135
190		PROCESS (M: ET-1, NO, leptin, fT3; +Covariates)	−0.0448 [−0.1194; 0.0299], p=0.238	−0.0079 [−0.0436; 0.0253]	Leptin −0.3795, p=0.0001***; BF% +0.0257, p=0.0001***; bSBP −0.0049, p=0.044*	0.135
198	**PWV**	Multivariate (HDL, age, sex, bSBP, BSA, BF%, MET-h)	+0.00010 [—], p=0.956		Age +0.060, p<0.001***; bSBP 0.030, p<0.001***	0.628 / 0.617
198		Multivariate (LDL, age, sex, bSBP, BSA, BF%, MET-h)	−0.002 [—], p=0.021		Age +0.056, p<0.001***; bSBP +0.031, p<0.001***	0.638 / 0.627
198		Multivariate (LDL/HDL, age, sex, bSBP, BSA, BF%, MET-h)	−0.051 [—], p=0.119		Age +0.061, p<0.001***; bSBP +0.031, p<0.001***	0.633 / 0.621
190		PROCESS (M: ET-1, NO, leptin, fT3 LDL; +Covariates)	+0.0011 [−0.0025; 0.0047], p=0.562	−0.0003 [−0.0015; 0.0008]	LDL −0.0018, p=0.021*; Age +0.054, p=0.0003***; bSBP 0.031, p<0.001***	0.650
190		PROCESS (M: ET-1, NO, leptin, fT3 HDL; +Covariates)	−0.0018 [−0.0032; −0.0003], p=0.0209	+0.0001 [−0.0003; 0.0006]	Age +0.054, p=0.0003***; bSBP 0.031, p<0.001***	0.650
190		PROCESS (M: ET-1, NO, leptin, fT3; +Covariates)	−0.0633 [−0.1294; 0.0029], p=0.0606	+0.0058 [−0.0089; 0.0285]	Age +0.056, p=0.0001***; bSBP 0.031, p<0.001***	0.646

*NO, nitric oxide; ET-1, endothelin-1; HDL, high-density lipoprotein; LDL, low-density lipoprotein; SRsys, systolic strain rate; SRdia, diastolic strain rate; PWV, pulse wave velocity; IMT, intima-media thickness; BF%, body fat percentage; MET-h, metabolic equivalent of task hours; BMI, body mass index; BSA, body surface area; MET, metabolic equivalent of task. Significant results are marked *p<0.05, **p<0.01, ***p<0.001.*

Table S3: Direct and indirect effects of fT3 on exercise performance and functional and structural vascular outcomes in young athletes.

N	Outcome	Model	Direct effect of fT3 (B, 95% CI), p	Indirect effects (PROCESS, B, 95%, Boot-CI)	Signif. Covariates (b, p)	R^2/adj. R^2
192	**VO2peak**	Multivariate (age, sex, FFM, MET-h)	+0.023 [−0.055; +0.101], p=0.562		FFM +0.047, p<0.001***; Age +0.066, p<0.001***; Sex +0.240, p<0.001***; MET-h +0.183, p=0.017*	0.905 / 0.902
190		PROCESS (M: ET-1, NO, HDL, LDL; Leptin +Covariates)	−0.005 [−0.0855; +0.0755], p=0.902	+0.0145 [−0.0246; +0.0488]	Age +0.0704, p<0.001***; Sex +0.1591, p=0.0004***; FFM +0.0466, p<0.001***	0.918
192	**Wattmax**	Multivariate (age, sex, FFM, MET-h)	+2.36 [−4.88; +9.60], p=0.524		FFM +4.075, p<0.001***; Age +9.56, p<0.001***; Sex +16.46, p<0.001***; MET-h +18.61, p=0.008**	0.908 / 0.906
190		PROCESS (M: ET-1, NO, HDL, LDL; Leptin +Covariates)	−0.467 [−7.757; +6.824], p=0.900	+1.566 [−2.289; +4.925]	Age +10.03, p<0.001***; Sex +9.64, p=0.016*; FFM +4.07, p<0.001***	0.922
192	**Strain**	Multivariate (age, sex, MET-h, bSBP)	+0.037 [−0.800; +0.874], p=0.931		Age +0.344, p=0.003**; Sex +1.552, p<0.001***	0.162 / 0.140
190		PROCESS (M: ET-1, NO, HDL, LDL; Leptin +Covariates)	+0.183 [−0.727; +1.093], p=0.692	+0.030 [−0.310; +0.361]	Age +0.319, p=0.017*; Sex +1.465, p=0.003**	0.200
192	**SRsys**	Multivariate (age, sex, MET-h, bSBP)	+0.033 [−0.059; +0.125], p=0.482		Age +0.026, p=0.046*	0.081 / 0.057
190		PROCESS (M: ET-1, NO, HDL, LDL; Leptin +Covariates)	+0.062 [−0.038; +0.162], p=0.223	−0.0067 [−0.0406; +0.0258]	–	0.115
192	**SRdia**	Multivariate (age, sex, MET-h, bSBP)	−0.067 [−0.169; +0.035], p=0.195		–	0.036 / 0.012
190		PROCESS (M: ET-1, NO, HDL, LDL; Leptin +Covariates)	−0.087 [−0.198; +0.023], p=0.121	+0.0189 [−0.0160; +0.0560]	–	0.064

192	**IMT**	Multivariate (age, sex, MET-h, BF%, bSBP)	−0.017 [−0.029; −0.005], p=0.007		Sex +0.020, p=0.002**	0.143 / 0.121
190		PROCESS (M: ET-1, NO, HDL, LDL; Leptin +Covariates)	−0.0204 [−0.0341; −0.0068], p=0.0035	+0.0014 [−0.0026; +0.0059]	Sex +0.0147, p=0.049*	0.178

*NO, nitric oxide; ET-1, endothelin-1; HDL, high-density lipoprotein; LDL, low-density lipoprotein; SRsys, systolic strain rate; SRdia, diastolic strain rate; PWV, pulse wave velocity; IMT, intima-media thickness; bSBP, systolic blood pressure; BF%, body fat percentage; FFM, fat free mass; MET-h, metabolic equivalent of task hours; BMI, body mass index; BSA, body surface area. Significant results are marked *p<0.05, **p<0.01, ***p<0.001.*

Table S4: Sex-specific differences in direct effects of NO, ET-1 and the NO-ET-1-ratio on functional and structural vascular outcomes in young athletes and an age-and sex-matched cohort.

**Outcome**	**N**	**Model**	**Direct effect (B, 95% CI)**	**p**	**R^2 / adj. R^2**	**Signif. covariates**
**Strain**	156 (M)	NO, unmatched	+1.881 [0.98; 2.79]	**<0.001*** **	0.194 / 0.168	-
	46-47 (F)		+1.586 [-0.29; 3.46]	0.105	0.124 / 0.015	-
	50 (M)	NO, matched	+2.918 [1.27; 4.57]	**0.001*** **	0.307 / 0.228	Age −0.884 (p=0.026*)
	46-47 (F)		+1.586 [-0.29; 3.46]	0.105	0.124 / 0.015	-
	156 (M)	ET-1, unmatched	+1.865 [0.95; 2.78]	**<0.001*** **	0.201 / 0.175	-
	46-47 (F)		+2.050 [0.08; 4.02]	**0.048***	0.139 / 0.032	-
	50 (M)	ET-1, matched	+2.888 [1.22; 4.56]	**0.002** **	0.291 / 0.210	Age −0.800 (p=0.048*)
	46-47 (F)		+2.050 [0.08; 4.02]	**0.048***	0.139 / 0.032	-
	156 (M)	NO/ET-1, unmatched	+1.872 [0.96; 2.79]	**<0.001*** **	0.194 / 0.167	-
	46-47 (F)		+1.824 [-0.03; 3.68]	0.060	0.157 / 0.052	-
	50 (M)	NO/ET-1, matched	+2.941 [1.28; 4.61]	**0.001*** **	0.294 / 0.213	Age −0.894 (p=0.028*)
	46-47 (F)		+1.824 [-0.03; 3.68]	0.060	0.157 / 0.052	-
**SRsys**	156 (M)	NO, unmatched	+0.108 [0.006; 0.210]	**0.040***	0.084 / 0.054	-
	46-47 (F)		+0.165 [-0.05; 0.33]	0.150	0.105 / -0.007	-
	50 (M)	NO, matched	0.241 [0.08; 0.40]	**0.005** **	0.210 / 0.120	Age −0.086 (p=0.025*)
	46-47 (F)		+0.165 [−0.05; 0.38]	0.150	0.105 / −0.007	-

	156 (M)	ET-1, unmatched	+0.107 [0.005; 0.209]	**0.041***	0.090 / 0.060	-
	46-47 (F)		+0.206 [-0.03; 0.44]	0.089	0.112 / 0.001	
	50 (M)	ET-1, matched	+0.242 [0.08; 0.40]	**0.005****	0.200 / 0.109	Age −0.085 (p=0.031*)
	46-47 (F)		+0.206 [−0.03; 0.44]	0.089	0.112 / 0.001	-
	156 (M)	NO/ET-1, unmatched	+0.107 [0.005; 0.209]	**0.042***	0.088 / 0.058	-
	46-47 (F)		+0.187 [-0.03; 0.41]	0.101	0.157 / 0.052	
	50 (M)	NO/ET-1, matched	+0.243 [0.08; 0.40]	**0.005****	0.209 / 0.119	Age −0.089 (p=0.023*)
	46-47 (F)		+0.187 [−0.03; 0.41]	0.101	0.126 / 0.017	-
**SRdia**	156 (M)	NO, unmatched	-0.088 [-0.20; 0.02]	0.137	0.054 / 0.022	-
	46-47 (F)		-0.143 [-0.39; 0.10]	0.259	0.127 / 0.018	-
	50 (M)	NO, matched	-0.231 [-0.40; -0.06]	**0.010***	0.234 / 0.147	Age +0.098 (p=0.016*)
	46-47 (F)		−0.143 [−0.388; 0.102]	0.259	0.127 / 0.018	-
	156 (M)	ET-1, unmatched	-0.085 [-0.20; 0.03]	0.146	0.054 / 0.023	-
	46-47 (F)		-0.184 [-0.45; 0.08]	0.184	0.069 / -0.047	-
	50 (M)	ET-1, matched	-0.227 [-0.40; -0.05]	**0.014***	0.177 / 0.084	Age +0.085 (p=0.043*)
	46-47 (F)		−0.184 [−0.451; 0.083]	0.184	0.069 / −0.047	-
	156 (M)	NO/ET-1, unmatched	-0.087 [-0.20; 0.03]	0.141	0.048 / 0.016	-
	46-47 (F)		-0.188 [-0.43; 0.05]	0.136	0.152 / 0.045	NO/ET-1 +0.444 (p=0.044*)
	50 (M)	NO/ET-1, matched	-0.235 [-0.41; -0.07]	**0.010***	0.200 / 0.109	Age +0.100 (p=0.017*)
	46-47 (F)		-0.188 [-0.43; 0.05]	0.136	0.152 / 0.045	NO/ET-1 +0.444 (p=0.044*)
**PWV**	156 (M)	NO, unmatched	+0.140 [0.040; 0.240]	**0.006****	0.629 / 0.617	bSBP +0.030 (p<0.001***)
	46-47 (F)		+0.125 [−0.093; 0.343]	0.269	0.631 / 0.585	bSBP +0.027 (p<0.001***)
	50 (M)	NO, matched	+0.117 [−0.050; 0.284]	0.179	0.674 / 0.637	bSBP +0.032 (p<0.001***)

	46-47 (F)		+0.125 [−0.093; 0.343]	0.269	0.631 / 0.585	bSBP +0.027 (p<0.001***)
	156 (M)	ET-1, unmatched	+0.140 [0.042; 0.238]	**0.006**	0.631 / 0.618	bSBP +0.030 (p<0.001***)
	46-47 (F)		+0.177 [−0.048; 0.402]	0.133	0.647 / 0.603	bSBP +0.028 (p<0.001***)
	50 (M)	ET-1, matched	+0.129 [−0.030; 0.288]	0.119	0.706 / 0.673	bSBP +0.031 (p<0.001***); ET-1 −0.698 (p=0.030*)
	46-47 (F)		+0.177 [−0.048; 0.402]	0.133	0.647 / 0.603	bSBP +0.028 (p<0.001***)
	156 (M)	NO/ET-1, unmatched	+0.141 [0.043; 0.239]	**0.006****	0.631 / 0.619	bSBP +0.030 (p<0.001***)
	46-47 (F)		+0.124 [−0.095; 0.343]	0.275	0.629 / 0.582	bSBP +0.027 (p<0.001***)
	50 (M)	NO/ET-1, matched	+0.122 [−0.041; 0.285]	0.152	0.690 / 0.655	bSBP +0.032 (p<0.001***)
	46-47 (F)		+0.124 [−0.096; 0.344]	0.275	0.629 / 0.582	bSBP +0.027 (p<0.001***)

*NO, nitric oxide; ET-1, endothelin-1; HDL, high-density lipoprotein; LDL, low-density lipoprotein; SRsys, systolic strain rate; SRdia, diastolic strain rate; PWV, pulse wave velocity; IMT, intima-media thickness; bSBP, systolic blood pressure; BF%, body fat percentage; MET-h, metabolic equivalent of task hours; BMI, body mass index; BSA, body surface area; MET, metabolic equivalent of task. Significant results are marked *p<0.05, **p<0.01, ***p<0.001.*

Table S5: Sex-specific differences in direct effects of LDL, HDL and the LDL-HDL-ratio on functional and structural vascular outcomes in young athletes and an age-and sex-matched cohort.

Outcome	N	Model	Direct effect (B, 95% CI)	p	R^2 / adj. R^2	Signif. covariates
**IMT**	152 (M)	HDL, unmatched	0.000 [n/a]	0.636	0.218 / 0.186	BSA +0.073 (p<0.001***); MET-h +0.037 (p=0.014*)
	46 (F)		+0.001 [−0.001; 0.003]	0.198	0.253 / 0.139	BSA +0.142 (p=0.002**)
	50 (M)	HDL, matched	+0.00005 [−0.002; 0.002]	0.932	0.130 / 0.008	-
	45 (F)		+0.001 [−0.001; 0.003]	0.180	0.267 / 0.151	BSA +0.145 (p=0.002**); Age −0.011 (p=0.046*)
	152 (M)	LDL, unmatched	−0.00001 [n/a]	0.933	0.217 / 0.184	BSA +0.075 (p<0.001***); MET-h +0.036 (p=0.016*)
	46 (F)		0.000 [n/a]	0.458	0.232 / 0.113	BSA +0.121 (p=0.008**)
	50 (M)	LDL, matched	0.000 [n/a]	0.444	0.141 / 0.022	-
	45 (F)		0.000 [n/a]	0.338	0.250 / 0.131	BSA +0.121 (p=0.007**); Age −0.012 (p=0.037*)

	152 (M)	LDL/HDL, unmatched	+0.002 [−0.008; 0.012]	0.690	0.218 / 0.185	BSA +0.075 (p<0.001***); MET-h +0.037 (p=0.015*)
	46 (F)		−0.017 [−0.039; 0.005]	0.143	0.263 / 0.149	BSA +0.126 (p=0.004**)
	50 (M)	LDL/HDL, matched	−0.007 [−0.032; 0.018]	0.586	0.135 / 0.015	-
	45 (F)		−0.019 [−0.041; 0.003]	0.095	0.286 / 0.174	BSA +0.128 (p=0.003**); Age −0.012 (p=0.034*)
**CD**	152 (M)	HDL, unmatched	0.000 [−0.006; 0.006]	0.871	0.153 / 0.118	BSA +0.637 (p<0.001***)
	46 (F)		+0.001 [−0.007; 0.009]	0.829	0.298 / 0.190	BSA +1.023 (p=0.004**)
	50 (M)	HDL, matched	+0.005 [−0.005; 0.015]	0.300	0.244 / 0.138	BSA +0.768 (p=0.023*)
	45 (F)		+0.001 [−0.007; 0.009]	0.837	0.299 / 0.188	BSA +1.020 (p=0.005**)
	152 (M)	LDL, unmatched	−0.001 [−0.003; 0.001]	0.450	0.156 / 0.121	BSA +0.612 (p<0.001***)
	46 (F)		+0.001 [−0.003; 0.005]	0.502	0.305 / 0.198	BSA +1.047 (p=0.003**)
	50 (M)	LDL, matched	−0.001 [−0.005; 0.003]	0.589	0.230 / 0.122	BSA +0.671 (p=0.043*)
	45 (F)		+0.001 [−0.003; 0.005]	0.478	0.307 / 0.198	BSA +1.046 (p=0.003**)
	152 (M)	LDL/HDL, unmatched	−0.021 [−0.117; 0.075]	0.662	0.154 / 0.119	BSA +0.632 (p<0.001***)
	46 (F)		+0.010 [−0.166; 0.186]	0.913	0.297 / 0.189	BSA +1.004 (p=0.004**)
	50 (M)	LDL/HDL, matched	−0.123 [−0.329; 0.083]	0.246	0.249 / 0.144	BSA +0.716 (p=0.029*)
	45 (F)		+0.013 [−0.167; 0.193]	0.888	0.298 / 0.187	BSA +1.002 (p=0.004**)
**cIDR**	152 (M)	HDL, unmatched	0.000 [n/a]	0.523	0.041 / 0.002	-
	46 (F)		0.000 [n/a]	0.235	0.177 / 0.051	-
	50 (M)	HDL, matched	−0.000074 [n/a]	0.517	0.025 / −0.111	-
	45 (F)		0.000 [n/a]	0.213	0.194 / 0.067	-
	152 (M)	LDL, unmatched	+0.000010 [n/a]	0.677	0.040 / 0.000	-
	46 (F)		−0.000048 [n/a]	0.293	0.171 / 0.043	BF% −0.001 (p=0.050*)
	50 (M)	LDL, matched	−0.000014 [n/a]	0.788	0.017 / −0.120	-
	45 (F)		−0.000059 [n/a]	0.197	0.197 / 0.070	-
	152 (M)	LDL/HDL, unmatched	+0.001 [−0.001; 0.003]	0.493	0.042 / 0.002	-

	46 (F)		−0.003 [−0.007; 0.001]	0.137	0.194 / 0.070	-
	50 (M)	LDL/HDL, matched	+0.001 [−0.005; 0.007]	0.739	0.018 / −0.119	-
	45 (F)		−0.004 [−0.008; 0.000]	0.088	0.223 / 0.100	-
**PWV**	152 (M)	HDL, unmatched	+0.002 [−0.002; 0.006]	0.242	0.646 / 0.631	bSBP +0.030 (p<0.001***); BSA +0.348 (p=0.012*)
	46 (F)		−0.004 [−0.012; 0.004]	0.269	0.627 / 0.570	bSBP +0.027 (p<0.001***)
	50 (M)	HDL, matched	+0.003 [−0.003; 0.009]	0.324	0.693 / 0.650	bSBP +0.032 (p<0.001***)
	45 (F)		−0.004 [−0.011; 0.004]	0.262	0.631 / 0.573	bSBP +0.027 (p<0.001***)
	152 (M)	LDL, unmatched	−0.002 [−0.004; −0.000]	0.027	0.654 / 0.640	bSBP +0.030 (p<0.001***); BSA +0.274 (p=0.042*)
	46 (F)		0.000 [−0.004; 0.004]	0.831	0.615 / 0.556	bSBP +0.027 (p<0.001***)
	50 (M)	LDL, matched	+0.001 [−0.003; 0.005]	0.592	0.688 / 0.644	bSBP +0.033 (p<0.001***)
	45 (F)		+0.001 [−0.003; 0.005]	0.744	0.620 / 0.560	bSBP +0.027 (p<0.001***)
	152 (M)	LDL/HDL, unmatched	−0.079 [−0.150; −0.009]	0.029	0.654 / 0.640	bSBP +0.030 (p<0.001***); BSA +0.317 (p=0.017*)
	46 (F)		+0.095 [−0.050; 0.240]	0.208	0.630 / 0.574	bSBP +0.026 (p<0.001***)
	50 (M)	LDL/HDL, matched	−0.010 [−0.163; 0.143]	0.895	0.686 / 0.642	bSBP +0.033 (p<0.001***)
	45 (F)		+0.104 [−0.043; 0.251]	0.173	0.637 / 0.580	bSBP +0.025 (p<0.001***)
**Strain**	152 (M)	HDL, unmatched	−0.003 [−0.040; 0.034]	0.863	0.240 / 0.209	BSA +5.939 (p<0.001***)
	46 (F)		−0.010 [−0.077; 0.057]	0.760	0.047 / −0.100	-
	50 (M)	HDL, matched	+0.009 [−0.054; 0.072]	0.768	0.391 / 0.306	Age −1.131 (p=0.004**); BSA +9.280 (p<0.001***)
	45 (F)		−0.009 [−0.074; 0.056]	0.793	0.056 / −0.093	-
	152 (M)	LDL, unmatched	−0.006 [−0.022; 0.010]	0.464	0.243 / 0.212	BSA +5.855 (p<0.001***)
	46 (F)		+0.008 [−0.021; 0.037]	0.619	0.050 / −0.096	-
	50 (M)	LDL, matched	−0.009 [−0.036; 0.018]	0.553	0.395 / 0.310	Age −1.119 (p=0.004**); BSA +8.968 (p<0.001***)
	45 (F)		+0.004 [−0.025; 0.033]	0.783	0.057 / −0.092	-
	152 (M)	LDL/HDL, unmatched	−0.032 [−0.679; 0.615]	0.923	0.240 / 0.209	BSA +5.983 (p<0.001***)
	46 (F)		+0.517 [−0.855; 1.889]	0.464	0.058 / −0.087	-

	50 (M)	LDL/HDL, matched	−0.472 [−1.907; 0.963]	0.522	0.396 / 0.311	Age −1.118 (p=0.004**); BSA +9.220 (p<0.001***)
	45 (F)		+0.383 [−0.980; 1.746]	0.585	0.062 / −0.086	-
**SRsys**	152 (M)	HDL, unmatched	0.000 [−0.004; 0.004]	0.919	0.110 / 0.074	BSA +0.375 (p=0.009**)
	46 (F)		−0.003 [−0.011; 0.005]	0.399	0.037 / −0.111	-
	50 (M)	HDL, matched	+0.002 [−0.004; 0.008]	0.494	0.277 / 0.176	Age −0.103 (p=0.008**); BSA +0.739 (p=0.002**)
	45 (F)		−0.003 [−0.011; 0.005]	0.415	0.062 / −0.086	-
	152 (M)	LDL, unmatched	−0.001 [−0.003; 0.001]	0.327	0.116 / 0.080	BSA +0.358 (p=0.012*)
	46 (F)		+0.001 [−0.003; 0.005]	0.509	0.030 / −0.119	-
	50 (M)	LDL, matched	0.000 [−0.002; 0.002]	0.806	0.270 / 0.168	Age −0.105 (p=0.007**); BSA +0.711 (p=0.003**)
	45 (F)		+0.001 [−0.003; 0.005]	0.723	0.048 / −0.102	-
	152 (M)	LDL/HDL, unmatched	−0.017 [−0.091; 0.057]	0.646	0.112 / 0.075	BSA +0.378 (p=0.008**)
	46 (F)		+0.103 [−0.058; 0.264]	0.216	0.057 / −0.088	-
	50 (M)	LDL/HDL, matched	−0.019 [−0.162; 0.124]	0.796	0.270 / 0.169	Age −0.104 (p=0.007**); BSA +0.708 (p=0.003**)
	45 (F)		+0.081 [−0.074; 0.236]	0.314	0.071 / −0.076	-
**SRdia**	152 (M)	HDL, unmatched	+0.002 [−0.002; 0.006]	0.490	0.093 / 0.055	BSA −0.432 (p=0.007**)
	46 (F)		+0.001 [−0.007; 0.009]	0.746	0.032 / −0.117	-
	50 (M)	HDL, matched	+0.001 [−0.005; 0.007]	0.842	0.252 / 0.148	Age +0.117 (p=0.005**); BSA −0.704 (p=0.006**)
	45 (F)		+0.001 [−0.007; 0.009]	0.762	0.029 / −0.124	-
	152 (M)	LDL, unmatched	0.000 [−0.002; 0.002]	0.895	0.090 / 0.052	BSA −0.457 (p=0.004**)
	46 (F)		−0.002 [−0.006; 0.002]	0.302	0.055 / −0.090	-
	50 (M)	LDL, matched	0.000 [−0.004; 0.004]	0.974	0.251 / 0.147	Age +0.117 (p=0.005**); BSA −0.715 (p=0.005**)
	45 (F)		−0.002 [−0.006; 0.002]	0.345	0.049 / −0.101	-
	152 (M)	LDL/HDL, unmatched	−0.035 [−0.117; 0.047]	0.407	0.094 / 0.056	BSA −0.454 (p=0.004**)
	46 (F)		−0.114 [−0.286; 0.058]	0.206	0.068 / −0.075	-
	50 (M)	LDL/HDL, matched	−0.009 [−0.164; 0.146]	0.911	0.252 / 0.147	Age +0.117 (p=0.005**); BSA −0.713 (p=0.005**)

	45 (F)		−0.109 [−0.285; 0.067]	0.236	0.062 / −0.086	-

*HDL, high-density lipoprotein; LDL, low-density lipoprotein; SRsys, systolic strain rate; SRdia, diastolic strain rate; PWV, pulse wave velocity; IMT, intima-media thickness; bSBP, systolic blood pressure; BF%, body fat percentage; MET-h, metabolic equivalent of task hours; BMI, body mass index; BSA, body surface area. Significant results are marked *p<0.05, **p<0.01, ***p<0.001.*

Table S6: Sex-specific differences in direct effects of fT3 on exercise performance and functional and structural vascular outcomes in young athletes and an age-and sex-matched cohort.

Outcome	N	Model	Direct effect (B, 95% CI)	p	R^2 / adj. R^2	Signif. covariates
**VO2peak**	152 (M)	fT3, unmatched	−0.015 [−0.102; 0.072]	0.735	0.912 / 0.910	Age +0.099, (p<0.001***); FFM +0.045, (p<0.001***)
	46 (F)		0.000 [−0.222; 0.222]	0.999	0.806 / 0.787	MET-h +0.421, (p<0.001***); FFM +0.044, (p<0.001***)
	50 (M)	fT3, matched	+0.109 [−0.034; 0.252]	0.135	0.934 / 0.928	Age +0.073, (p=0.016*); FFM +0.050, (p<0.001***)
	45 (F)		−0.089 [−0.309; 0.131]	0.417	0.833 / 0.816	MET-h 0.369, (p=0.001***); FFM 0.044, (p<0.001***)
**Wattmax**	152 (M)	fT3, unmatched	−0.538 [−8.866; 7.790]	0.899	0.910 / 0.907	Age +12.252, (p<0.001***); FFM +3.877, (p<0.001***)
	46 (F)		−3,649 [−22.293; 14.995]	0.695	0.854 / 0.840	MET-h +28.499, (p=0.004**); FFM +4.208, (p<0.001***)
	50 (M)	fT3, matched	+11.200 [−4.65; 27.05]	0.162	0.908 / 0.900	Age +12.076, (p<0.001***); FFM +4.009, (p<0.001***)
	45 (F)		−10.160 [−28.88; 8.56]	0.279	0.870 / 0.857	MET-h +24.697, (p=0.010*); FFM +4.201, (p<0.001***)
**Strain**	152 (M)	fT3, unmatched	+0.021 [−0.90; 0.95]	0.963	0.235 / 0.209	FFM +0.123, (p<0.001***)
	46 (F)		+0,131 [−2.08; 2.35]	0.906	0.050 / −0.069	-
	50 (M)	fT3, matched	−0,889 [−2.61; 0.83]	0.303	0.374 / 0.303	Age −0.903, (p=0.011*); FFM +0.189, (p<0.001***)
	45 (F)		+0.723 [−1.55; 3.00]	0.524	0.071 / −0.048	-
**SRsys**	152 (M)	fT3, unmatched	+0.032 [−0.073; 0.137]	0.545	0.115 / 0.085	FFM +0.008, (p=0.004**)
	46 (F)		0.002 [−0.261; 0.265]	0.986	0.018 / −0.105	-
	50 (M)	fT3, matched	−0.050 [−0.221; 0.121]	0.560	0.252 / 0.167	Age −0.086, (p=0.015*); FFM +0.015, (p=0.001***)
	45 (F)		0.099 [−0.164; 0.362]	0.450	0.057 / −0.064	-
**SRdia**	152 (M)	fT3, unmatched	−0.078 [−0.197; 0.041)]	0.196	0.079 / 0.047	FFM −0.008, (p=0.015*)
	46 (F)		−0.042 [−0.327; 0.243]	0.769	0.021 / −0.101	-
	50 (M)	fT3, matched	+0.014 [−0.173; 0.201]	0.878	0.212 / 0.122	Age +0.093, (p=0.015*); FFM −0.015, (p=0.004**)

	45 (F)		−0.078 [−0.379; 0.223]	0.606	0.025 / −0.099	-
PWV	152 (M)	fT3, unmatched	+0.027 [−0.076; 0.130]	0.602	0.640 / 0.627	bSBP +0.031, (p<0.001***); FFM +0.006, (p=0.023*)
	46 (F)		−0.103 [−0.342; 0.136]	0.389	0.616 / 0.568	bSBP +0.027, (p<0.001***)
	50 (M)	fT3, matched	0.026 [−0.157; 0.209]	0.774	0.677 / 0.640	bSBP +0.032, (p<0.001***)
	45 (F)		−0.143 [−0.394; 0.108]	0.258	0.625 / 0.576	bSBP +0.027, (p<0.001***)
IMT	152 (M)	fT3, unmatched	−0.017 [−0.031; −0.003]	**0.015**	0.227 / 0.195	MET-h +0.030, (p=0.045*); FFM +0.001, (p<0.001***)
	46 (F)		−0.044 [−0.080; −0.008]	**0.018**	0.306 / 0.199	Age −0.019, (p=0.006**); FFM +0.003, (p=0.003**)
	50 (M)	fT3, matched	−0.025 [−0.053; 0.003]	0.092	0.182 / 0.068	-
	45 (F)		−0.041 [−0.079; −0.003]	**0.038**	0.293 / 0.181	Age −0.019, (p=0.007**); FFM +0.003, (p=0.003**)

*fT3, free triiodothyronine; SRsys, systolic strain rate; SRdia, diastolic strain rate; PWV, pulse wave velocity; IMT, intima-media thickness; bSBP, systolic blood pressure; BF%, body fat percentage; FFM, fat free mass; MET-h, metabolic equivalent of task hours. Significant results are marked *p<0.05, **p<0.01, ***p<0.001.*

Table S7: Sex-specific differences in direct effects of leptin on endothelial and functional and structural vascular outcomes in young athletes and an age-and sex-matched cohort.

Outcome	N	Model	Direct effect (B, 95% CI)	p	R^2 / adj. R^2	Signif. covariates
NO	147 (M)	Leptin, unmatched	+0.095 [−0.008; 0.198]	0.069	0.053 / 0.026	BF% −0.009 (p=0.020*)
	45 (F)		+0.258 [−0.035; 0.551]	0.083	0.091 / 0.000	-
	46 (M)	Leptin, matched	+0.204 [−0.034; 0.442]	0.092	0.087 / −0.002	-
	45 (F)		+0.258 [−0.035; 0.551]	0.083	0.091 / 0.000	-
Strain	147 (M)	Leptin, unmatched	+1.479 [−0.235; 3.193]	0.091	0.137 / 0.106	BF% −0.137 (p=0.028*)
	45 (F)		+0.831 [−2.714; 4.376]	0.638	0.052 / −0.070	-
	46 (M)	Leptin, matched	+1.841 [−2.141; 5.823]	0.356	0.182 / 0.080	-
	45 (F)		+0.831 [−2.715; 4.377]	0.638	0.052 / −0.070	-

**SRsys**	147 (M)	Leptin, unmatched	+0.167 [−0.021; 0.355]	0.080	0.087 / 0.054	-
	45 (F)		−0.098 [−0.503; 0.307]	0.626	0.051 / −0.071	-
	46 (M)	Leptin, matched	+0.185 [−0.185; 0.555]	0.318	0.133 / 0.024	-
	45 (F)		−0.098 [−0.503; 0.307]	0.626	0.051 / −0.071	-
**SRdia**	147 (M)	Leptin, unmatched	−0.418 [−0.616; −0.220]	**<0.001*****	0.147 / 0.117	BF% +0.026 (p<0.001***)
	45 (F)		+0.127 [−0.336; 0.590]	0.582	0.034 / −0.090	-
	46 (M)	Leptin, matched	−0.226 [−0.616; 0.164]	0.248	0.144 / 0.037	-
	45 (F)		+0.127 [−0.336; 0.590]	0.582	0.034 / −0.090	-
**PWV**	147 (M)	Leptin, unmatched	+0.118 [−0.060; 0.296]	0.191	0.638 / 0.625	bSBP +0.031 (p<0.001***); Age +0.067 (p<0.001***)
	45 (F)		+0.276 [−0.117; 0.669]	0.164	0.613 / 0.563	bSBP +0.028 (p<0.001***)
	46 (M)	Leptin, matched	+0.079 [−0.275; 0.433]	0.653	0.672 / 0.631	bSBP +0.034 (p<0.001***); Age +0.082 (p=0.004**)
	45 (F)		+0.276 [−0.117; 0.669]	0.164	0.613 / 0.563	bSBP +0.028 (p<0.001**)
**IMT**	147 (M)	Leptin, unmatched	−0.012 [−0.036; 0.012]	0.352	0.123 / 0.092	Age +0.005 (p=0.022*)
	45 (F)		+0.050 [−0.015; 0.115]	0.128	0.097 / −0.019	-
	46 (M)	Leptin, matched	−0.058 [−0.117; 0.001]	0.055	0.152 / 0.046	-
	45 (F)		+0.050 [−0.015; 0.115]	0.128	0.097 / −0.019	-

*NO, nitric oxide; SRsys, systolic strain rate; SRdia, diastolic strain rate; PWV, pulse wave velocity; IMT, intima-media thickness; bSBP, systolic blood pressure; BF%, body fat percentage; MET-h, metabolic equivalent of task hours. Significant results are marked *p<0.05, **p<0.01, ***p<0.001.*
